# 84 Predicting Intra-Operative Blood Product Requirements During Burn Surgery

**DOI:** 10.1093/jbcr/irae036.083

**Published:** 2024-04-17

**Authors:** Evan L Barrios, Jeremy Balch, John Rodriguez, Mariami Gogolishvili, Josef Ali, Burn Unit, Alicia Y Burris, Burn Unit, Andrea Munden, Amalia Cochran, Ian R Driscoll

**Affiliations:** University of Florida, Gainesville, Florida; University of Florida, Gainesville, Florida; University of Florida, Gainesville, Florida; University of Florida, Gainesville, Florida; University of Florida, Gainesville, Florida; University of Florida, Gainesville, Florida; University of Florida, Gainesville, Florida; University of Florida, Gainesville, Florida; University of Florida, Gainesville, Florida; University of Florida, Gainesville, Florida; University of Florida, Gainesville, Florida

## Abstract

**Introduction:**

Burn patients require significant blood products during their hospitalization. Blood is a critical and limited resource. Up to one-third of blood products are transfused intraoperatively. Prior research identified amount of tissue excised, hemoglobin, INR, and platelets as predictive of transfusions of fresh frozen plasma (FFP) and red blood cells (RBCs). However, development of a model based on both transfused and non-transfused patients intraoperatively has not been performed. Predicting a patient’s likelihood of requiring a transfusion, as well as describing how much blood product to type-and-cross preoperatively, will result in improved utilization of a limited resource.

**Methods:**

This retrospective analysis examined 254 burn operations representing 104 unique patients between October 2019-August 2020 at a single burn unit. Initial model features included age, TBSA, 2nd/3rd degree burn, ICU/hospital length of stay, preoperative hemoglobin, amount of tissue excised/grafted, case length, if case was a re-excision, and total blood transfused. Few missing variables were imputed using Multi-imputation by Chained Equation. We first built a multiclass logistic regression model for units of RBCs and FFP. Variables with the strongest coefficients were preoperative hemoglobin, case length, amount second-degree burn, and amount excised. Four models were assessed for performance metrics: index cases (the first operation for each patient) and FFP, index cases and RBCs, full cohort (including re-operations) and FFP, full cohort and RBCs. Given poor discrimination at higher levels of transfusion, we then applied linear regression on a subset of patients who received transfusions.

**Results:**

Models had accuracy ranges from 0.56 (full cohort and FFP) to 0.76 (index cohort and RBC). Models had excellent precision, recall, and f1 scores in predicting patients not any requiring transfusions, with declining performance at higher transfusion levels. Interestingly, machine learning models (Random forest, XGBoost, SVM, Decision Tree) had similar accuracy ranges compared to logistic regression. With respect to linear regression performed on the transfused patient cohort, R^2 values were 0.43 and 0.31 for RBCs and FFP, respectively. RBC and FFP transfusion requirements (mL) can be broadly estimated using the following three variables:

RBC_ml = [Anticipated Area Excised (cm^2 ) × 0.1] + [Anticipated Case Length (min) × 6.2] - [Hgb_Preop × 70] + 350

FFP_mL = [Anticipated Area Excised (cm^2 ) × 0.1] + [Anticipated Case Length (min) × 3] - [Hgb_Preop × 35] + 275

**Conclusions:**

This modeling allows for moderate accuracy in type-and-cross predictions preoperatively, with excellent accuracy in predicting the need for a transfusion overall. Increased sample size may permit greater model predictive ability using machine learning techniques.

**Applicability of Research to Practice:**

Increased accuracy in preoperative type-and-cross improves blood resource utilization.

